# The phyllosphere microbiome shifts toward combating melanose pathogen

**DOI:** 10.1186/s40168-022-01234-x

**Published:** 2022-04-02

**Authors:** Pu-Dong Li, Zeng-Rong Zhu, Yunzeng Zhang, Jianping Xu, Hongkai Wang, Zhengyi Wang, Hongye Li

**Affiliations:** 1 The Key Laboratory of Molecular Biology of Crop Pathogens and Insects of Ministry of Agriculture, The Key Laboratory of Biology of Crop Pathogens and Insects of Zhejiang Province, Institute of Biotechnology, Zhejiang University, 866 Yuhangtang Road, Hangzhou, 310058 China; 2 Hainan Institute, Zhejiang University, Sanya, 572000 China; 3 Joint International Research Laboratory of Agriculture and Agri-Product Safety, The Ministry of Education of China, Yangzhou University, Yangzhou, 225009 China; 4 Department of Biology, McMaster University, 1280 Main St. West, Hamilton, ON L8S 4K1 Canada

**Keywords:** Phyllosphere microbiota, Citrus melanose, *Diaporthe citri*, High-throughput sequencing, Disease suppression

## Abstract

**Background:**

Plants can recruit beneficial microbes to enhance their ability to defend against pathogens. However, in contrast to the intensively studied roles of the rhizosphere microbiome in suppressing plant pathogens, the collective community-level change and effect of the phyllosphere microbiome in response to pathogen invasion remains largely elusive.

**Results:**

Here, we integrated 16S metabarcoding, shotgun metagenomics and culture-dependent methods to systematically investigate the changes in phyllosphere microbiome between infected and uninfected citrus leaves by *Diaporthe citri*, a fungal pathogen causing melanose disease worldwide. Multiple microbiome features suggested a shift in phyllosphere microbiome upon *D*. *citri* infection, highlighted by the marked reduction of community evenness, the emergence of large numbers of new microbes, and the intense microbial network. We also identified the microbiome features from functional perspectives in infected leaves, such as enriched microbial functions for iron competition and potential antifungal traits, and enriched microbes with beneficial genomic characteristics. Glasshouse experiments demonstrated that several bacteria associated with the microbiome shift could positively affect plant performance under *D*. *citri* challenge, with reductions in disease index ranging from 65.7 to 88.4%. Among them, *Pantoea* asv90 and *Methylobacterium* asv41 identified as “recruited new microbes” in the infected leaves, exhibited antagonistic activities to *D*. *citri *both in vitro and in vivo, including inhibition of spore germination and/or mycelium growth. *Sphingomonas* spp. presented beneficial genomic characteristics and were found to be the main contributor for the functional enrichment of iron complex outer membrane receptor protein in the infected leaves. Moreover, *Sphingomonas* asv20 showed a stronger suppression ability against *D*. *citri* in iron-deficient conditions than iron-sufficient conditions, suggesting a role of iron competition during their antagonistic action.

**Conclusions:**

Overall, our study revealed how phyllosphere microbiomes differed between infected and uninfected citrus leaves by melanose pathogen, and identified potential mechanisms for how the observed microbiome shift might have helped plants cope with pathogen pressure. Our findings provide novel insights into understanding the roles of phyllosphere microbiome responses during pathogen challenge.

**Video abstract**

**Supplementary Information:**

The online version contains supplementary material available at 10.1186/s40168-022-01234-x.

## Background

Like the rhizosphere, the phyllosphere (aboveground part of terrestrial plants) is an important niche of the plant [1, 2]. The phyllosphere is inhabited by diverse microbes, with some microbes living on the surface of plants as epiphytes and others colonizing inside tissues (e.g., leaves) as endophytes [3]. These microbes form microbial communities that are associated with phyllosphere habitats. The microbial communities associated with the phyllosphere are collectively called the phyllosphere microbiome [4].

Since the colonization of land by ancestral plant lineages 450 million years ago, plants have been intricately linked with microbes and the fitness of each plant is a consequence of the interactions between the plant and its microbiome, which collectively form a holobiont [5, 6]. Recent studies have shown that changes in plant microbiome are not merely a passive response by plants but rather, as a consequence of coevolution, plants are likely to actively seek cooperation with microbes to relieve stresses [7]. In particular, plants can employ the “cry for help” strategy to enhance their ability to combat stresses. For instance, plants experiencing biotic or abiotic stresses recruited beneficial microbes/traits from the environment [8, 9]. Such plant-microbe interactions are considered to be critical for maintaining plant health [10]. In recent years, there has been increasing evidence for rhizosphere-mediated resistance to pathogen invasion and for maintaining plant health through regulation of the microbiome. For example, members of the Chitinophagaceae and Flavobacteriaceae families were enriched in the root endosphere of sugar beet during invasion by the fungal pathogen *Rhizoctonia solani* and that the reconstruction of a consortium of *Chitinophaga* and *Flavobacterium* consistently suppressed *R*. *solani*-induced root disease [11]. Similarly, successive wheat plantings and infection by *R*. *solani* shape the rhizosphere microbiota and specifically accumulate a group of beneficial microbes, such as *Chitinophaga*, *Pseudomonas*, *Chryseobacterium*, and *Flavobacterium* [12]. In addition, transplantation of rhizosphere microbiota from *Ralstonia solanacearum* disease-resistant tomato cultivar suppressed *R*. *solanacearum* disease development in susceptible tomato cultivar [13], with the competition for iron as a potential mechanism for control against the pathogen *R*. *solanacearum* by the natural rhizosphere microbiomes in tomato plants [14]. However, whether and how the phyllosphere microbiomes respond to pathogen challenge for the benefits of the plant hosts are not well understood. Uncovering the relationship between pathogen invasion and phyllosphere microbiome response at the collective community-level could help us better understand the microbe-plant-pathogen interactions, and provide additional evidence to support the “cry for help” strategy in the plant holobiont.

*Diaporthe citri* is one of the most destructive fungal pathogens of citrus, which induces multiple symptoms including melanose on leaves, fruits and shoots, stem-end rot of fruits, gummosis or blight of perennial branches and trunks [15–17]. *D*. *citri* has a worldwide distribution, and all citrus species and cultivars are susceptible to its infection, including mandarin, tangerine, sweet orange, pomelo, grapefruit, and lemon [18]. During its life cycle, *D*. *citri* saprophytically colonizes the dead wood of citrus for the majority of time, and it grows and produces conidia and ascospores there, with both spores provide the inoculums for melanose disease [19]. Chemical fungicides are the main agents for managing melanose disease worldwide [20]. The phyllosphere microbiome represents a potential line of defense against pathogens. A clear understanding of how the phyllosphere microbiome responds to *D*. *citri* infection can help us develop better strategies to prevent and control melanose disease.

In this study, we integrated 16S metabarcoding, shotgun metagenomic sequencing and culture-dependent approaches to investigate and compare the phyllosphere microbiomes between *D*. *citri*-infected and uninfected leaves from citrus trees under natural field conditions. The aim of this study was to (i) describe the shift in the phyllosphere microbiome between infected and uninfected leaves, (ii) investigate whether and how members of the phyllosphere microbiome shift conferred positive effects on the plant upon pathogen attack, and (iii) provide additional evidence in the phyllosphere niche to support the “cry for help” strategy in plants.

## Results

### Phyllosphere microbiota structure and diversity differed between infected and uninfected leaves

**Fig. 1:**
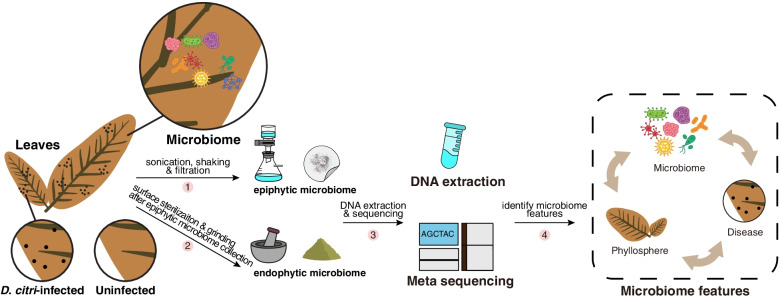
Microbiome feature identifications of citrus leaves naturally infected/uninfected by *Diaporthe citri*. The infected and uninfected samples were collected from a citrus orchard in August 2019, where the melanose disease (caused by *D*. *citri*) had been severe in the three preceding years. A workflow for separation, DNA extraction, and sequencing of the phyllosphere microbiomes

To explore variation in the phyllosphere microbiota between *D*. *citri*-infected and uninfected conditions, we collected the leaf samples from a citrus orchard in August 2019 in Quzhou, Zhejiang province, China, where the melanose disease had been severe in the three preceding years (Fig.1 and Additional file 1: Fig. S1-2). We generated a microbial community profile for each sample using 16S metabarcoding data (Additional file 2: Table S1). In total, we obtained 3,109,915 sequences from 40 phyllosphere samples. After denoising, removing low-quality and chimeric sequences with DADA2 [21], amplicon sequence variants (ASVs) were obtained from epiphytic and endophytic bacteria, with 2590 and 472 ASVs respectively.

**Fig. 2:**
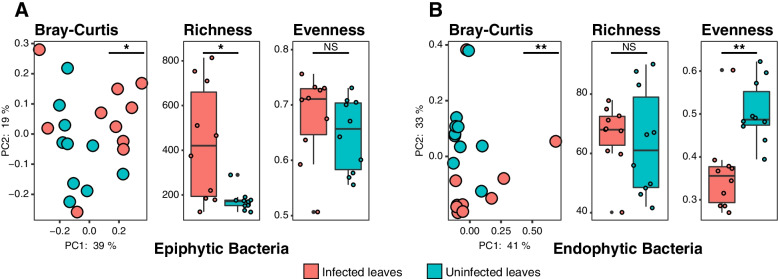
Community structure and microbial diversity between infected and uninfected phyllosphere microbiotas. The different patterns of phyllosphere microbiotas in **a** epiphytic and **b** endophytic phyllosphere. Unconstrained PCoA (for principal coordinates PCo1 and PCo2) with Bray-Curtis metrics was conducted to characterize the beta diversity and analyzed in PERMANOVA to test for differences. Richness (observed ASVs) and Pielou’s Evenness indices were conducted to characterize the alpha diversity and analyzed in Kruskal-Wallis to test for differences. Asterisks denote significant differences (*P < 0.05; **P < 0.01; ***P < 0.001) and NS denotes no statistical significance

Our results showed that the phyllosphere microbiotas have different patterns of community structure and microbial diversity between the epiphytic and endophytic compartment niches (Fig. 2 and Additional file 2: Table S2). PCoA indicated that the community structure between the infected and uninfected leaves differed significantly in both the epiphytic (PERMANOVA; *P* < 0.05) (Fig. 2a) and endophytic phyllosphere (PERMANOVA; *P* < 0.01) (Fig. 2b). Infected leaves had greater microbial richness for epiphytic bacteria (Kruskal-Wallis; *P* < 0.05) than those on uninfected leaves (Fig. 2a). Within the endophytic bacteria, no significant difference in microbial richness was found between the infected and uninfected leaves (Fig. 2b). However, the community evenness of endophytic bacteria in the infected leaves was significantly lower (Kruskal-Wallis; *P* < 0.01) than that in the uninfected leaves (Fig. 2b).

**Fig. 3:**
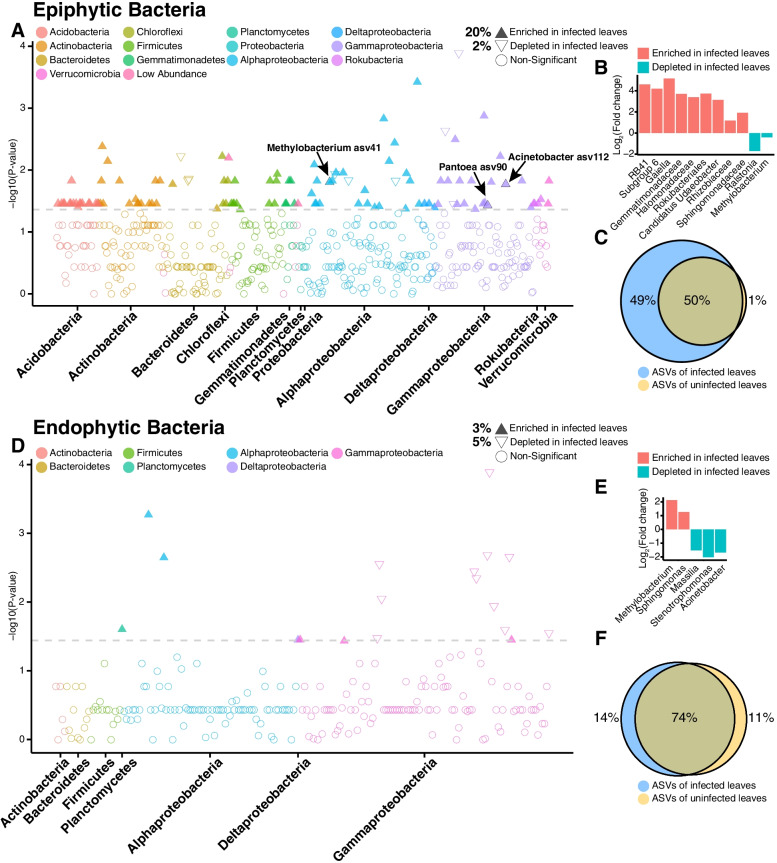
Taxonomic characteristics of differential bacteria between infected and uninfected phyllosphere microbiotas. **a**, **d** Manhattan plots showing ASVs enriched or depleted in the infected samples in phyllosphere microbiota. Each circle or triangle represents a single ASV. ASVs enriched or depleted in the infected samples are represented by filled or empty triangles, respectively (ASVs abundance > 0.01%, *P* < 0.05). **b**, **e** The enriched or depleted genus (if not annotated to specific genera, using higher-level taxa to represent) of the infected samples in the phyllosphere (taxa abundance > 0.1%, *P* < 0.05). Data bars represent the log2 fold-change. **c**, **f** Venn diagram depicting the number of ASVs identified in phyllosphere microbiota from the infected and uninfected samples

Comparison of microbiotas in infected leaves to those in uninfected leaves identified more differential ASVs in the epiphytic compartment were those that showed enriched distributions (Fig. 3a). In the endophytic compartment, the number of enriched ASVs was slightly lower than the depleted ASVs (Fig. 3d). These are consistent with the results of the analysis of variance at the genus level (Fig. 3b, e). Moreover, the epiphytic bacteria presented a large number of distinct ASVs (new microbes) unique to infected samples (Fig. 3c), which were distinctly different from endophytic bacteria (Fig. 3f). Overall, 109 bacterial ASVs were enriched in the infected epiphytic leaves. Among them, *Methylobacterium* asv41, *Pantoea* asv90, and *Acinetobacter* asv112 represented the “abundant new microbes” in infected leaves (abundance > 0.1%), which were present in ≥ 60% infected samples and in ≤ 20% uninfected samples (Fig. 3a and Additional file 2: Table S3).

### Phyllosphere microbiota shows intense microbial interactions in infected leaves

To explore whether changes in microbial community assemblies (Figs. 2 and 3) were accompanied by changes in microbial interactions, we performed co-occurrence network analysis and estimated the topological properties to uncover potential associations and complexity of connections among the phyllosphere microbiota.

**Fig. 4:**
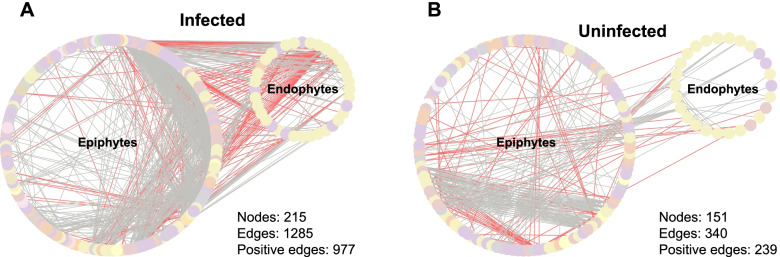
Co-occurrence networks between infected and uninfected phyllosphere microbiotas. Network inference was performed based on SparCC correlation at ASVs level (abundance > 0.01%) to compare the microbial interactions between **a** infected and **b** uninfected conditions. Each node represents a single ASV, which was colored based on class level. A connection stands for a statistically significant (*P* < 0.05) correlation with magnitude > 0.7 (positive correlation, gray edges) or < − 0.7 (negative correlation, red edges)

Co-occurrence network analysis revealed increased connectivity and complexity in the infected condition compared with the uninfected condition in the phyllosphere (Fig. 4). Specifically, we observed that the increased connectivity in infected leaves was largely attributed to interactions from the epiphytic compartment, including epiphytes-epiphytes and epiphytes-endophytes associations (Fig. 4a). Our topological analysis of networks provided further insights into the variation of interactions (Additional file 2: Table S4). The network of the infected leaves presented a higher number of connections per node (average degree = 11.95) than that in uninfected leaves (4.50), indicating a highly connected microbial community. A similar result was observed when nodes from the endophytic compartment were removed, and the average degree of network in infected and uninfected leaves was 10.84 and 4.41, respectively.

### Shotgun metagenomic data provide deeper insights into the microbial interactions in the phyllosphere microbiome

Multiple microbiome features from metabarcoding analyses suggested a shift in phyllosphere microbiome upon challenge with *D*. *citri* (Figs. 2, 3, 4). Overall, epiphytic bacteria presented more complicated variations than endophytic bacteria, in both microbial enrichment (Fig. 3a, d) and microbial network relationships (Fig. 4). To further explore the role of epiphytic bacteria in phyllosphere microbiome from the functional perspective, we performed shotgun metagenomic sequencing (Additional file 2: Table S5). The combined datasets of the infected and uninfected leaf samples contained a total of 133.8 Gbp of raw reads. Quality filtering and removal of plant-derived reads resulted in 79.4-Gbp microbial reads. In total, 7,621,585 putative protein-coding genes were predicted from the assembly. After clustering sequences at 95% identity, 5,592,809 non-redundant genes were generated.

**Fig. 5:**
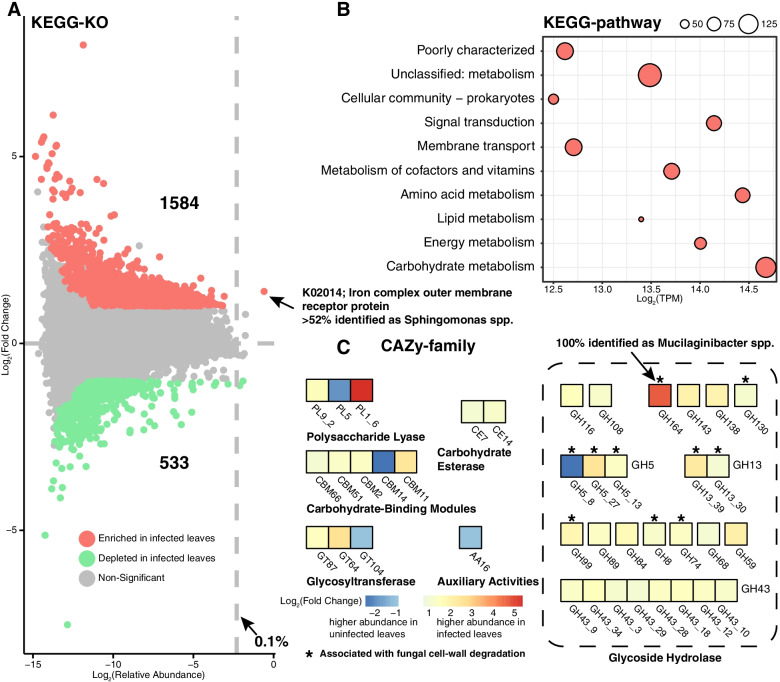
Overview of functional profiling of the epiphytic microbiome. **a** Enrichment and depletion of the KOs, fold change > 2 or < − 2, respectively. **b** The top 10 pathways with enriched-KOs in the infected samples involved in KEGG pathway. The circle sizes represent the number of enriched-KOs. The *X*-axis represents the abundance of genes (TPM, log2 transformed) involved in pathway. **c** Diversity and distribution of carbohydrate-active enzymes (CAZy) in the epiphytic microbiome. Differential abundance analysis revealed that 40 CAZy families significantly changed between infected and uninfected samples (DESeq2; *P* < 0.05, FDR < 0.1)

Of the genes retrieved from the shotgun metagenomic data, 58% were assigned to a known function. Functional annotations were obtained for 53% non-redundant genes by aligning to the KEGG database and generated 15,423 KOs (KEGG Orthologs, GhostKOALA Score > 10). Our results showed that the epiphytic microbiome in the infected leaves possessed significantly higher KO diversity (Kruskal-Wallis; *P* < 0.05) than those in the uninfected leaves (Additional file 1: Fig. S3). Differential enrichment analysis (DESeq2; fold change > 2 or < − 2, *P* < 0.05, FDR < 0.1) of the epiphytic microbiomes between the infected and uninfected leaves revealed that 1584 KOs were enriched in the infected samples, whereas 533 KOs were depleted (Fig. 5a). Furthermore, we found that most of the enriched KOs belonged mainly to metabolism-related pathways. Specifically, a large number of enriched KOs were involved in pathways of unclassified-metabolism (173 KOs), carbohydrate metabolism (136 KOs), metabolism of cofactors and vitamins (92 KOs), amino acid metabolism (83 KOs), energy metabolism (63 KOs), and lipid metabolism (44 KOs), which together represent 6 of top 10 pathways with enriched KOs (Fig. 5b and Additional file 2: Table S6). Remarkably, K02014 (iron complex outer membrane receptor protein) was the most abundant differentially enriched KO (Fig. 5a), at 2.6-fold higher in infected leaves compared to uninfected leaves. The majority of the K02014 genes were associated with *Sphingomonas* spp. K02014, consisting of 14,165 contigs from our shotgun metagenomic data where > 52% of which were annotated as *Sphingomonas* spp. This suggested that the competition for iron of *Sphingomonas* spp. may play a role in the microbiome-pathogen interactions in the phyllosphere.

For more resolution of specific functions associated with metabolism-related pathways, we searched for carbohydrate-active enzymes (CAZy) database within the shotgun metagenomic data (Additional file 2: Table S7). Like KO diversity, our results also showed that the epiphytic microbiome in the infected leaves possessed significantly higher CAZy diversity (Kruskal-Wallis; *P* < 0.05) than those in the uninfected leaves (Additional file 1: Fig. S3). Differential abundance analysis revealed that 40 CAZy families significantly (DESeq2; *P* < 0.05, FDR < 0.1) changed between the infected and uninfected samples of the epiphytic microbiomes (Fig. 5c). Among them, 35 CAZy gene families were more abundant in the infected samples than those in the uninfected samples. Remarkably, the microbiome of infected leaves showed a greater functional capacity for glycoside hydrolase than the microbiome of uninfected leaves. Many of these glycoside hydrolase families may be associated with the degradation of fungal cell wall components [22], including glucans (GH5, GH13, and GH74), mannans (GH5, GH99, GH130, and GH164), and chitosan (GH5 and GH8). Among these, the GH164 gene family (β-mannosidase) showed the largest change in abundance, with a 25.3-fold increase in the infected samples compared to the uninfected samples. The GH164 family genes were located on two contigs in our assembled shotgun metagenomic data, with both contigs annotated as *Mucilaginibacter* spp.

### Metagenome-assembled genomes reveal functions of *Sphingomonas* spp.

**Fig. 6:**
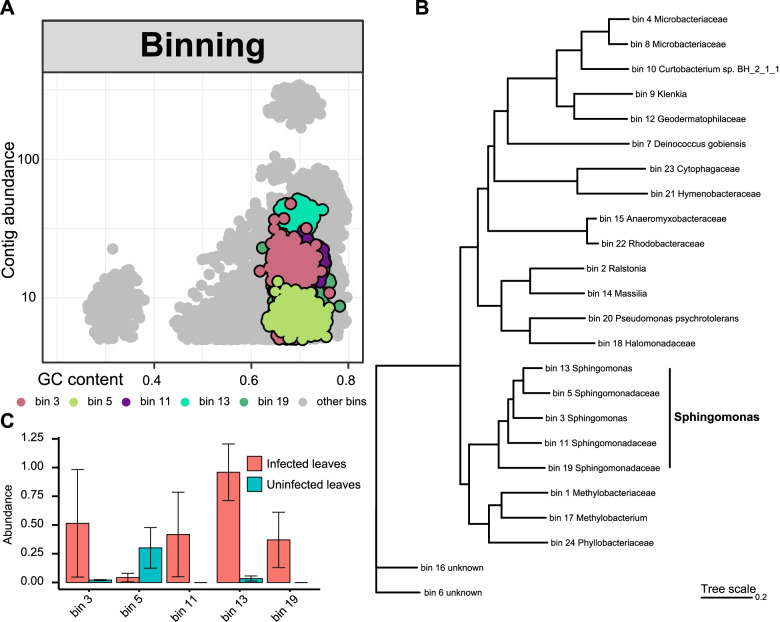
Binning of the shotgun metagenomic data and reconstruction of the genomes. **a** Scatterplot representing the distribution of the assembled contigs based on their GC content and abundance. **b** Phylogenetic tree of the draft genomes (bins, *n* = 24) from the MAGs (metagenome assembled genomes) data (completeness > 70%, contamination < 10%). The tree was produced from concatenated alignments of up to 400 ubiquitously conserved proteins identified with PhyloPhlAn and used the SGB (species-level genome bins) release to assign to each genome bin its closest SGB. Genome bin 3, 5, 11, 13, and 19 were confidently assigned to the *Sphingomonas* genus using a higher-resolution phylogenetic tree (Additional file 1: Fig. S4). **c** The abundance (genome copies per million reads) of the *Sphingomonas* bins in the infected and uninfected samples

Next, we attempted to explore the microbial functions in the epiphytic phyllosphere from a genomic perspective (Additional file 2: Table S8). The draft genomes were binned using the metagenome-assembled genome (MAG) extraction approach (Fig. 6a). After assembly, 24 bins (draft genomes) had completeness > 70% and contamination < 10%. Phylogenetic analysis demonstrated that five Sphingomonadaceae bins clustered into a clade (Fig. 6b). We selected 272 Sphingomonadaceae representative genomes and the Sphingomonadaceae bins (in this study) to further construct a high-resolution phylogenetic tree, which demonstrated that these Sphingomonadaceae bins were confidently assigned to the *Sphingomonas* genus (Additional file 1: Fig. S4).

Among these five *Sphingomonas* bins, bin 13 had the highest quality (completeness = 90.52%, contamination = 7.46%, N50 = 16,164). The assembled genome was comprised of 260 contigs with a total length of 2.62 Mb. Genomic analysis indicated that these *Sphingomonas* bins have multiple plant-beneficial characteristics, such as IAA production (e.g., *trpA*, *trpB*, *trpC*, and *trpD*), sulfur assimilation (e.g., *cysQ*, *cysW*, *cysT*, and *cysA*), phosphate metabolism (e.g., *pstA*, *pstC*, *phoU*, *phoR*, *pstB*, and *pstS*), and niche colonization (chemotaxis) (e.g., *CheA*, *CheW*, and *CheR*) (Additional file 2: Table S9-13). Four of these five *Sphingomonas* bins (bin 3, 11, 13, and 19) were more abundant in infected leaves compared to uninfected leaves (Fig. 6c). These metagenomic results are consistent with our metabarcoding data (Additional file 2: Table S3), and suggest a potential beneficial role for these *Sphingomonas* microbes in the phyllosphere following *D*. *citri* infection. It should be noted that not all *Sphingomonas* bins were enriched in the infected leaves compared to the uninfected leaves. For example, bin 5 also contained beneficial genomic characteristics; however, its abundance was higher in uninfected leaves than in infected leaves (Fig. 6c and Additional file 2: Table S10). Moreover, multiple genes of *Sphingomonas* bins were classified as K02014 (iron complex outer membrane receptor protein) functional category, with up to 36 genes, similar to the results from direct metagenomic functional annotation (Fig. 5a and Additional file 2: Table S9-13).

### Microbes as part of the phyllosphere microbiome shift could positively affect plant performance against *D. citri* invasion

**Fig. 7:**
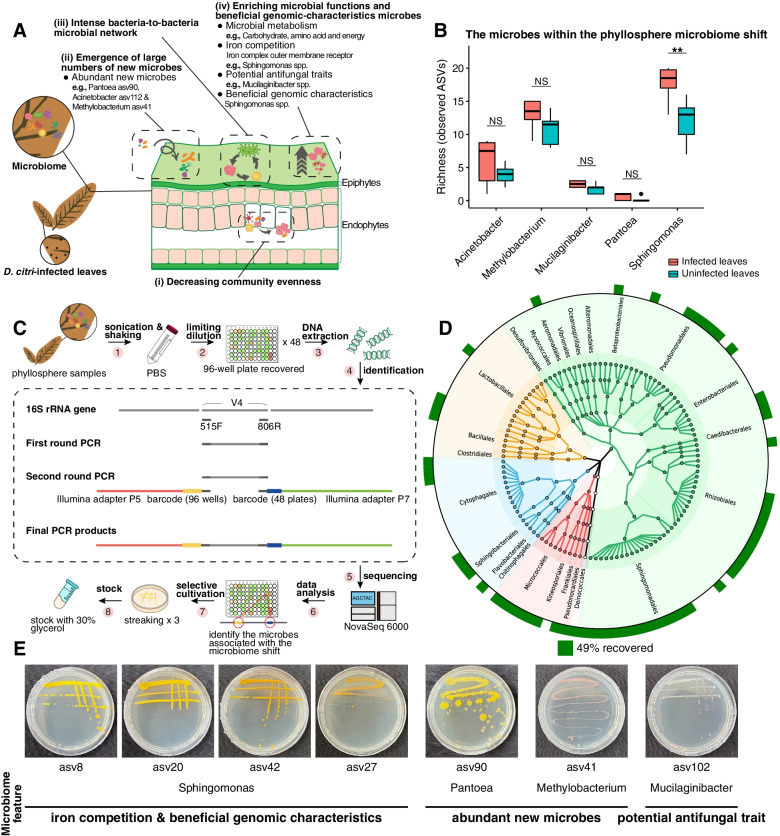
Microbes associated with the phyllosphere microbiome shift*.* **a** Overview of microbiome features in citrus leaves infected by *D*. *citri*. (**i–iii**) Microbiome features suggested the shift in phyllosphere microbiome upon challenge with *D*. *citri*. **b** Richness (observed ASVs) of the microbes within the phyllosphere microbiome shift from 16S metabarcoding data. Asterisks denote significant differences (*P < 0.05; **P < 0.01; ***P < 0.001) and NS denotes no statistical significance. **c** A workflow for microbial cultivation of the microbes within the phyllosphere microbiome shift. **d** Culture-dependent coverage (48.7%) of epiphytic phyllosphere-associated bacteria. The inner ring represents the epiphytic phyllosphere-associated ASVs reproducibly detected from 16S metabarcoding data (with relative abundance greater than 0.1%). The outer ring with green squares represents ASVs that are founded in the cultivated bacteria derived from epiphytic phyllosphere. **e** Twelve microbes associated with the phyllosphere microbiome shift were selectively cultivated, belonging to the genera *Sphingomonas* (9 ASVs), *Pantoea* (asv90), *Methylobacterium* (asv41), and *Mucilaginibacter* (asv102). Four (asv8, asv20, asv42, and asv27) of 9 *Sphingomonas* ASVs were used for further study because of their antagonistic activities against *D*. *citri*

From microbiome sequencing data, we evaluated the changes of phyllosphere microbiome following *D*. *citri* infection, and identified multiple microbiome features within the phyllosphere microbiome shift (Fig. 7a, b). We further investigated the effects of the microbes within phyllosphere microbiome shift on plant performance upon *D*. *citri* challenge. We adopted a two-step barcoded PCR method [23, 24] of the recovered bacteria in combination with Illumina NovaSeq 6000 to target the same 16S rDNA V4 region as the metabarcoding data of the epiphytic bacteria (Fig. 7c). Comparing the V4 region sequence identity of these two datasets allows us to selectively cultivate bacteria at the ASV level. In total, 249 unique bacteria with distinct ASVs were recovered from 96-well plates, and accounted for 48.7% bacterial ASVs (relative abundance > 0.1%) that we identified in our corresponding 16S metabarcoding data (Fig. 7d). Of these, we selectively cultivated 12 bacteria associated with the phyllosphere microbiome shift (Fig. 7a), including 9 *Sphingomonas* (9 ASVs), 1 *Pantoea* (asv90), 1 *Methylobacterium* (asv41), and 1 *Mucilaginibacter* (asv102) (Fig. 7e).

**Fig. 8:**
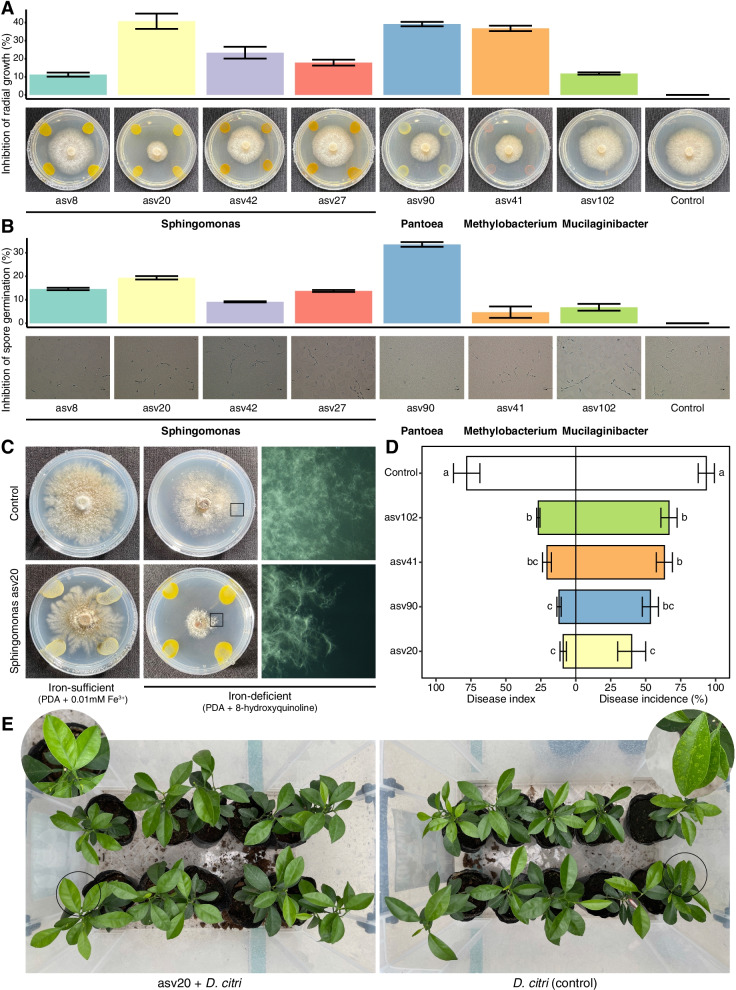
Suppressive activities of selected microbes from the changed component of the phyllosphere microbiome against the melanose pathogen *D*. *citri*. **a** The antagonistic activities in dual culture assays. A mycelial plug of *D*. *citri* was placed in the center of the medium, and tested isolates were streaked on four ends. Plates inoculated with *D*. *citri* only served as control. **b** Effect of microbial metabolites on *D*. *citri* spore germination in vitro. The *D*. *citri* spore suspension spread on water agar medium supplemented with 10% cell-free supernatant of tested isolates. The spore suspension spread on water agar medium supplemented with 10% 1/2 TSB medium served as control. **c** Effect of iron nutrition on antagonistic activity of *Sphingomonas* asv20 against *D*. *citri* in dual culture assays. **d** Disease suppression of melanose in the phyllosphere treated with *Sphingomonas* asv20, *Pantoea* asv90, *Methylobacterium* asv41, and *Mucilaginibacter* asv102 in glasshouse experiments. Non-treated seedlings were used as controls. Disease index and disease incidence were scored at 10 days after *D*. *citri* inoculation. Different letters indicate statistically significant differences between treatments as determined by one-way ANOVA and LSD test (*P* < 0.05). **e** Pictures showing citrus seedling leaves treated/untreated with *Sphingomonas* asv20 at 10 days after *D*. *citri* inoculation

These bacteria were then tested in dual culture assays for their antagonism against melanose pathogen *D*. *citri*. Seven of the 12 bacterial isolates exhibited different antagonistic activities against *D*. *citri* (Fig. 8a). *Sphingomonas* asv20, *Pantoea* asv90, and *Methylobacterium* asv41 showed strong antagonistic activities, and the percent inhibition of radial growth values were 40.8 ± 4.2%, 39.2 ± 1.2%, and 36.8 ± 1.5%, respectively. Furthermore, we also examined the inhibitory activities in vitro using metabolites from these microbial strains (Fig. 8b). We observed that the metabolites of *Pantoea* asv90 exhibited the marked reduction of spore germination, and the percent inhibition of spore germination was 33.5 ± 1.0%.

The microbiome feature identified from shotgun metagenomic data mentioned above suggests that the competition for iron of *Sphingomonas* may play a role in the microbiome-pathogen interactions in the phyllosphere (Fig. 5a). We selected a strain (asv20) with the most antagonistic capacity among *Sphingomonas* spp. to investigate whether iron conditions affect its antagonistic activity (Fig. 8c). When antagonism was assessed on iron-deficient and iron-sufficient media by dual culture method, suppression of *D. citri* mycelial growth was observed. *Sphingomonas* asv20 showed significantly higher suppression of mycelial growth in the iron-deficient medium than in the iron-sufficient medium (ANOVA; *P* < 0.001), indicating that iron limitation enhanced the pathogen-suppression effect of *Sphingomonas* asv20.

The above bacterial isolates were further tested to determine their disease suppression activity against *D*. *citri* in the phyllosphere in glasshouse experiments. The leaves of citrus seedlings were treated with *Sphingomonas* asv20, *Pantoea* asv90, *Methylobacterium* asv41, and *Mucilaginibacter* asv102, respectively, before inoculation with *D*. *citri*. Overall, treatment with these microbes conferred protection against *D*. *citri* infection as compared with the non-treated control at different levels, with the reduction in both disease incidence (ranging from 26.7 to 40.0%) and disease index (ranging from 65.7 to 88.4%) (Fig. 8d, e).

Taken together, these results suggested that several microbes within the shifted phyllosphere microbiome could improve plant performance against *D*. *citri* invasion.

## Discussion

The microbiome has long been recognized as an essential part of the crop ecosystem and is closely linked to the growth of plants and resistance to diseases [9]. There is growing evidence that plants experiencing biotic or abiotic stress can use a range of chemical stimuli to recruit beneficial microbes/traits from the environment to enhance their ability to combat the stress [7]. This phenomenon in which plants actively seeking cooperation with microbes to combat stresses is known as the “cry for help” strategy [8]. In recent years, the response of rhizosphere microbiome (underground part) in maintaining plant health has been extensively studied [11–13], while the phyllosphere microbiome (aboveground part) has been less studied. In this study, we profiled the changes in phyllosphere microbiome between infected and uninfected citrus leaves by melanose pathogen *D*. *citri*, and underlined the phyllosphere microbiome shift that could help plants cope with the pathogen attack. Our findings advance the understanding of the role of phyllosphere microbiome responses upon pathogen challenge.

Microbiome changes have been described across many plant species upon pathogen challenge, such as huanglongbing in citrus [25], common scab in potato [26], and crown rot disease in wheat [27]. Changes in the microbiome that associated with improved or reduced plant performance under stressful conditions [28, 29]. These studies emphasized that the microbiome response could play an important role in maintaining plant health. Aside from analyzing changes in microbial taxa, changes in microbial interactions based on network inferences can provide additional clues to the impact of microbiome changes on plant health. Our study here revealed that the phyllosphere microbiota of infected leaves presented the more intense bacterial-to-bacterial microbial network than those of uninfected leaves (Figs. 4 and 7a). Highly connected networks, like those in the infected leaves, can occur when microbes face environmental pressures, such as pathogen invasion [30]. Previous studies have also indicated that the complicated network could reduce pathogen invasion success [31]. Thus, our results suggested that a potentially beneficial role for the intense microbial networks observed in infected leaves. Similar to epiphytic microbiota, endophytic microbiota has also been studied as a second microbiological layer of plant defense [32]. However, the role of the community evenness in the endophytic microbiota in response to pathogen invasion has not been reported. Our data demonstrated that the great reduction of community evenness in infected leaves compared to uninfected leaves (Figs. 2b and 7a), implying that only a limited number of microbiota members in the endophytic phyllosphere may involve in the response to *D*. *citri* infection.

Large numbers of new microbes (at ASV level) emerge in the infected leaves compared to the uninfected leaves (Figs. 3c and 7a). These new ASVs may confer new microbial functions to the native microbiota in response to *D*. *citri* invasion. Results from functional diversity analyses of the phyllosphere microbiome also support this view (Additional file 1: Fig. S3). *Pantoea* asv90 and *Methylobacterium* asv41 were widely present in infected samples and less present in uninfected samples (Figs. 3a and 7a). Both bacteria exhibited antagonistic activities against the melanose pathogen *D*. *citri* (Fig. 8a, b). Therefore, they can be considered the “recruited new microbes”. Emerging evidence demonstrated that many recruited microbes following stresses, can be beneficial for plant survival. For example, *Arabidopsis thaliana* recruited a group of disease resistance-inducing and growth-promoting beneficial microbes upon downy mildew pathogen *Hyaloperonospora arabidopsidis* infection [33]. The wheat plant recruited *Stenotrophomonas rhizophila* SR80 to suppress soil-borne pathogens [27]. Sugar beet roots enriched for Chitinophagaceae and Flavobacteriaceae in the root endosphere, which contributed to the antifungal phenotype [11]. It should be noted that the recruited microbes shown in these studies were enriched from the native microbiota. Our study emphasized that the phyllosphere microbiome response presented a joint contribution of both the native microbes (e.g., *Sphingomonas* spp.) and the recruited new microbes (e.g., *Pantoea* asv90 and *Methylobacterium* asv41) when challenged by *D*. *citri*.

Multiple functional categories related to microbial metabolism were enriched in the infected leaves (Figs. 5b and 7a), consistent with the intense microbial network after *D. citri* infection (Fig. 4). This result suggested that the plant phyllosphere may enhance the metabolism-based interactions involving microbe-microbe and/or plant-microbe interactions following *D*. *citri* challenge. In addition, the epiphytic phyllosphere enriched microbial functions for iron competition (i.e., iron complex outer membrane receptor protein) upon *D*. *citri* invasion (Figs. 5a and 7a). Remarkably, although different microbial taxa can provide functionally redundant features to a plant host [28, 34], *Sphingomonas* spp. were found to be the main contributor for the functional enrichment of this microbiome feature. Previous studies have indicated that *Sphingomonas* species possess multifaceted functions ranging from remediation of environmental contaminations to promoting plant growth [35]. The *Sphingomonas* microbes found in our study also exhibited multiple genomic characteristics that are beneficial for plants (Additional file 2: Table S9-13). However, few studies have reported the contribution of *Sphingomonas* to alleviate infection by plant pathogens or other biotic stress. A recent study found that *Sphingomonas melonis* confers resistance to disease-susceptible phenotypes in rice seeds [36]. Our evidence suggested that *Sphingomonas* suppressed melanose pathogen possibly through competition for iron in the phyllosphere (Figs. 5a and 8c-e). Genomic functional annotations suggested that this competition may be achieved through TonB-dependent transport systems (Additional file 2: Table S9-13). The production of a large number of TonB-dependent transport systems by *Sphingomonas* strains might confer strong environmental adaptation in a nutrient-poor phyllosphere [1, 37], which may explain their antagonistic effects based on competition for iron. A previous study revealed the important role of microbial competition for iron in maintaining plant health by rhizosphere microbiomes [14]. However, evidence for iron competition-mediated disease suppression by phyllosphere microbiomes has not been reported. It is entirely predictable that the competition for scarce resources to confer protection to plant against pathogen invasion may work better in the nutrient-poor phyllosphere than in the nutrient-rich rhizosphere. *Sphingomonas* was among the most common bacterial taxa in the plant phyllosphere, not only on the model plant *A*. *thaliana*, but also on many other plant species [38–42]. However, whether *Sphingomonas* also acts as a defense line of the phyllosphere on other systems of plant-pathogen interaction requires further investigation. A recent study showed that *Sphingomonas* exhibited high diversity in the phyllosphere across many plant species [43], which was similar to our results (Fig. 7b). It needs to be noted that the infected leaves showed significantly higher (Wilcoxon test; *P* < 0.01) Richness (observed ASVs) of *Sphingomonas* spp. than the uninfected leaves (Fig. 7b). However, the exact contribution of this variation to plant adaptation to disease stress remains to be determined. Our results also demonstrated that several potential antifungal traits (associated with degradation of fungal cell wall components) were enriched in infected leaves (Figs. 5c and 7a), suggesting that the functional response of phyllosphere microbiome might be involved in fungal pathogen suppression. This view was supported by a previous study, which demonstrated that the pathogen invasion activated the enrichment of chitinase genes in the endophytic root microbiome to suppress the fungal pathogen [11].

Collectively, our evidence demonstrates that when challenged by *D*. *citri*, the phyllosphere microbiome shift reflects a targeted response by the plants to alleviate pathogen stress through regulating microbiome interactions and structure.

## Conclusions

Overall, our findings demonstrated that after melanose pathogen infection, several members within the changed component of the phyllosphere microbiome exerted beneficial effects on the citrus host. The phyllosphere microbiome shift presented a joint contribution of the native microbes and the recruited new microbes. Among the changed microbes, *Sphingomonas* showed significant potential to confer protection to the citrus phyllosphere against pathogen invasion through its iron-competition ability. Our study provides novel insights for understanding the roles of phyllosphere microbiome responses during pathogen challenge and their effect on plant health, and provides phyllosphere evidence to support the “cry for help” strategy in plants.

## Methods

### Sample collection and DNA extraction

Two groups of leaf samples (*D*. *citri*-infected and uninfected) of *Citrus unshiu* (> 20 years old), commonly known as tangerine and Satsuma orange, were collected from an orchard in August 2019 in Quzhou (29° 5′ 38″ N, 119° 2′ 16″ E), Zhejiang province, China. The infected and uninfected leaves were identified based on visual symptoms and qPCR results (Additional file 1: Fig. S1-2). A total of 6–8 leaves (infected/uninfected) per tree were collected and represented one replicate, placed in sterile plastic bags, and immediately transported to the laboratory and stored at − 80 °C pending further processing. In total, > 150 leaves from ten trees (replicates) of the two groups were used in this study.

To collect the epiphytic microbes, leaves (from each of the replicate) were first transferred into a 250-mL conical flask containing 200 mL 0.01 M sterile phosphate-buffered saline (PBS, 4 °C), and then subjected to sonication (15 min) and shaking (1 h, 200 rpm, 20 °C) to dislodge the epiphytic microbes from the leaf surface into the washing solution. The washing solution was filtered with a 0.22-μm clean sterile cellulose membrane filter to capture all the microbes in the solution. The membrane was cut into pieces (2 × 2 mm) with sterilized scissors [44] (Fig. 1).

To collect the endophytic microbes, the leaf samples washed using the procedure described above were surface sterilized by consecutive immersion for 1 min in 75% ethanol, 3 min in 1% sodium hypochlorite, and 30 s in 75% ethanol, followed by three rinses with sterile water. Subsequently, the treated leaves were freeze-dried and homogenized [44] (Fig. 1).

The leaf samples treated above (epiphytic or endophytic) were subjected to DNA extraction using the FastDNA® Spin Kit for Soil (MP Biomedical, Solon, OH, USA) according to the manufacturer’s instructions. The DNA quality was investigated by running the extracted DNA on a 1.0% agarose gel and the DNA concentrations were measured using a NanoDrop 1000 spectrophotometer (Thermo Scientific). In total, four sample types were prepared, epiphytic and endophytic microbes from infected and uninfected leaves, respectively. For each of the four sample types, ten biological replicates were prepared for metabarcoding sequencing, and three biological replicates for shotgun metagenomic sequencing (Fig. 1).

### DNA metabarcoding and shotgun metagenomic sequencing

For targeted metabarcoding sequencing, DNA fragments were amplified using primers targeting the 16S rDNA V4 region for bacteria (Additional file 2: Table S1). PCR reaction was done for each sample under the following conditions [45]: 30 s at 98 °C; 35 cycles of denaturation at 98 °C for 10 s, annealing at 54 °C for 30 s, and extension at 72 °C for 45 s; final step 10 min at 72 °C. The 25 μL of PCR mixture contained 12.5 μL of Phusion® High-Fidelity PCR Master Mix (New England Biolabs Inc., Beverly, MA), 2.5 μL of each primer (1 μM), and 50 ng of template DNA. The PCR products were electrophoresed and stained with ethidium bromide for visualization. The specific DNA bands on the gel were excised and purified using Qiagen Gel Extraction Kit (Qiagen, Germany). Sequencing libraries were generated using TruSeq® DNA PCR-Free Sample Preparation Kit (Illumina, USA) following the manufacturer’s recommendations and then sequencing was conducted using the Illumina NovaSeq 6000 Sequencer (Illumina, Inc., CA, USA).

For shotgun metagenomic sequencing, the extracted DNA samples were fractionated by ultrasound into approximately 350 bp fragments. The fragments were then used to construct the sequencing libraries using NEBNext® Ultra™ DNA Library Prep Kit for Illumina (NEB, USA). The libraries were sequenced using the Illumina NovaSeq 6000 Sequencer (Illumina, Inc., CA, USA).

### Metabarcoding data processing and co-occurrence network inferences

For metabarcoding data, the reads were analyzed using QIIME 2 (Quantitative Insights Into Microbial Ecology 2, version 2019.7) and its plugins [46]. Briefly, to obtain amplicon sequence variants (ASVs), we first used DADA2 to remove low-quality reads and noises, including removing chimeras [21]. The ASVs were then assigned to taxonomy groupings based on comparisons with the Silva database [47] using the q2-feature-classifier [48]. Alpha diversity indices (Richness and Pielou's Evenness) and beta diversity metrics (Bray-Curtis) were calculated through the diversity plugin (q2-diversity).

For network analyses, co-occurrence networks of the samples were performed using the SparCC [49] correlation calculated by FastSpar [50] to assess the complexity of the interactions in phyllosphere microbiota [51]. For each network, P-values were obtained by 100 permutations of random selections of the original ASV count data. The high relative abundances (> 0.01%) and statistically significant correlations (*P* < 0.05, SparCC correlations with a magnitude > 0.7 or < − 0.7) among ASVs were included into the network analyses. To analyze the topology of the co-occurrence networks, we used a set of measures such as the numbers of edges, nodes and clusters, clustering coefficients, modularity, connectance, average degree, network diameter, and average path length. Network visualization was carried out in Cytoscape [52].

### Shotgun metagenomic assembly and annotation

For the shotgun metagenomic data, the raw reads were trimmed with the sliding window approach to generate the QC (Quality Control) reads using Trimmomatic [53]. Contamination of reads originating from the host plant were aligned to the nuclear genome of *Citrus unshiu* (GCA_002897195.1) [54], the plastid genomes of *Citrus sinensis* (NC_008334.1) [55], and *Citrus maxima* (NC_034290.1), and the mitochondrial genome of *Citrus sinensis* (NC_037463.1) [56] using Bowtie 2 [57]. After that, the concordantly mapped reads were removed to preserve the clean reads. To obtain the microbial reads and their taxonomic annotations, the clean reads were aligned against the NCBI nr (non-redundant) database [58] using Kraken 2 [59], and the reads that could not be aligned to bacteria, fungi, archaea or virus were filtered out. The microbial reads from all the samples were pooled together for de novo assembly with MEGAHIT [60]. For the resulting contigs, ORF (Open Reading Frame) were predicted with Prodigal [61] in metagenomics mode and the non-redundant gene categories were generated using CD-HIT [62] with an identity cutoff of 95%. For gene annotation, DIAMOND [63] was used to annotate with nr database [58] and SWISS-PROT database [64]. For functional annotation, GhostKOALA [65] and DIAMOND were used to annotate with the KEGG database [66] and CAZy (carbohydrate-active enzymes) database [67], respectively. Reads counts and TPM (transcripts per million) of each predicted gene were determined with Salmon [68].

### Genome binning and genomics analysis

Assembled metagenomic data were binned with MetaBAT 2 [69], MaxBin 2 [70], and CONCOCT [71]. Refinement and reassemble steps were then performed using the MetaWRAP [72] pipeline to combine and improve the results generated by the three binners (completion > 70% and contamination < 10%). We determine the taxonomy of each bin using Taxator-tk [73] against the NCBI nt database [58]. The bins were then quantified by Salmon [68] with default parameters. A phylogenetic tree of bins was constructed using PhyloPhlAn [74] based on concatenated alignments of up to 400 ubiquitously conserved proteins and used the SGB (species-level genome bins) release [75] to assign to each genome bin its closest SGB. The thresholds of Mash distance we used to assign taxonomic labels were 0.05, 0.15, and 0.30 for species, genus, and families, respectively [75].

The genome bins were further purified prior to genomic analysis. Briefly, RefineM identify and remove potential contamination based on the genomic properties (GC, tetranucleotide signatures, coverage) of contigs and based on their taxonomic assignment against the Genome Taxonomy Database (GTDB) [76]. Sphingomonadaceae representative genomes and the Sphingomonadaceae bins were selected to further construct a high-resolution phylogenetic tree using PhyloPhlAn [74]. The genome bins were predicted the protein-coding gene using Prodigal [61] with default parameters. For functional annotation, eggNOG-mapper were used to annotate with eggNOG database [77].

### Bacterial isolation from the epiphytic phyllosphere

The PBS washing solution containing the epiphytic microbes was prepared according to the method mentioned above. Then, the washing solution was empirically diluted, distributed, and recovered in 96-well microtiter plates in 1/10 TSB medium for 16 days at 28 °C. We adopted a two-step barcoded PCR protocol [23, 24] in combination with Illumina NovaSeq 6000 to target the 16S rDNA V4 region (same region as 16S metabarcoding mentioned above) of the epiphytic bacteria. Positions of isolates in 96-well microtiter plates were indexed by two-step PCR primers containing well- and plate-specific barcodes to amplify the variable V4 region. Recovered bacteria were compared with ASVs in corresponding epiphytic microbiota members (16S metabarcoding) with > 99% 16S rRNA gene identity to calculate the culture-dependent coverage. Each unique ASVs identified from recovered bacteria were selectively cultivated and purified on the 1/2 TSA medium. These isolates were used for validation by Sanger sequencing with both 27F and 1492R primers.

### In vitro and in vivo antagonistic activities of bacteria against *D*. *citri*

The antagonistic activities of phyllosphere bacteria were tested by in vitro dual culture assays. A mycelial plug of *D*. *citri* (isolated from the infected samples, and used in the following experiments) was placed in the center of 1/2 TSA medium. Tested isolates were streaked on four ends of the plate and cultured at 26 °C for 7 days to examine antagonistic activities in vitro. Plates inoculated with *D*. *citri* only served as controls, and three replicates were performed. In three independent biological experiments, the percent inhibition of radial growth was calculated as follows: 100 × [(R1 − R2)/R1], where R1 was the radial growth of *D*. *citri* in the control and R2 was the radial growth of *D*. *citri* in the dual culture assays.

In vitro spore germination inhibition assays were performed using cell-free supernatant of the tested strains. Bacterial cells were collected from 4-day-old liquid cultures (1/2 TSB medium) by centrifugation for 10 min at 10,000×g. The resulting supernatant was then filtered through a 0.22-μm filter to remove the cells. Spore germination (%) was evaluated following 13 h incubation at 26 °C of 50 μL *D*. *citri* spore suspension (2 × 10^6^ CFU/mL) spread on water agar medium supplemented with 10% of each of the cell-free supernatant. Spores with germ tubes at least half the length of the spore were considered germinated. The spore suspension spread on water agar medium supplemented with 10% 1/2 TSB medium served as controls, and three replicates were performed. In three independent biological experiments, the percent inhibition of spore germination was calculated as follows: (G1 − G2)/G1, where G1 was the percentage of control spore germination and G2 was the percentage of supernatant-addition spore germination.

To investigate the effect of iron nutrition on the antagonistic activity of *Sphingomonas* against *D*. *citri*, an in vitro test for antagonism of *Sphingomonas* was done under iron-deficient and iron-sufficient conditions against *D*. *citri*. The methodology for antagonism study was performed as mentioned above. PDA medium containing 0.01 mM FeCl_3_ was iron-sufficient medium, whereas PDA medium treated with 10 mg/L 8-hydroxyquinoline (an iron chelating agent) was considered iron-deficient medium.

The in vivo antagonistic activities of bacteria against *D*. *citri* were investigated in glasshouse experiments. Citrus seedlings (*Citrus reticulata* cv. Hongjv) were grown in the commercial substrate (Klasmann-Deilmann, Germany) in a glasshouse with a 28/20 °C day/night temperature regime and natural sunlight. Tested isolates were cultivated in 1/2 TSB medium for 2 days at 28 °C on a rotary shaker at 180 rpm. Liquid cultures (adjusted to 10^8^ CFU/mL) of each isolate were centrifuged, washed 3 times, and resuspended in sterile 10 mM MgCl_2_. Approximately 1 mL of cell suspension was sprayed on each seedling (45-day-old). Non-treated seedlings (treated with MgCl_2_) were used as controls. After 2 days, seedling leaves were inoculated with approximately 1 mL of conidial suspension of *D*. *citri* (2 × 10^5^ CFU/mL). For each independent treatment, ten replicates were used. In three independent biological experiments, disease incidence and disease index were assessed at 10 days after pathogen inoculation. The disease index was rated on a scale of: 0 = no lesions, 1 = 1 to 5 lesions, 3 = 6 to 10 lesions, 5 = 11 to 15 lesions, 7 = 16 to 20 lesions, 9 = more than 20 lesions.

### Statistical analysis

All statistical analyses were performed using specific packages in R version 3.6.3 (The R Foundation for Statistical Computing, Vienna, Austria) [78].

Kruskal-Wallis test was used to compare the significant difference of alpha diversity. PERMANOVA (Permutational Multivariate Analysis of Variance) was used to examine whether the sample groups harbored significantly different microbial community (i.e., beta diversity). Wilcoxon test was used to identify the statistical significance in differences of the relative abundance at different taxa between the groups. At the functional level, differentially abundant KOs (KEGG Orthologs) and CAZy gene families were identified using DESeq2 [79]. Statistically significant differences between inoculation-treatments were determined by one-way ANOVA and LSD test.

## Supplementary Information

**Additional file 1: Supplementary Figures. **This additional file contains 4 supplementary figures, referred to in the main text.

**Additional file 2: Supplementary Tables. **This additional file contains 13 supplementary tables, referred to in the main text.

## Data Availability

All the raw sequencing data from this project are available in the NCBI Sequence Read Archive (SRA) database under BioProject PRJNA643596.
