# Supplementary material for: The phyllosphere microbiome shifts toward combating melanose pathogen
Source: Microbiome. 2022 Apr 2;10:56. doi: 10.1186/s40168-022-01234-x (PMC8976405; doi:10.1186/s40168-022-01234-x)
Supplement: Supplementary file 2 — Additional file 1: Supplementary Figures. This additional file contains 4 supplementary figures, referred to in the main text. [file 40168_2022_1234_MOESM2_ESM.pdf]

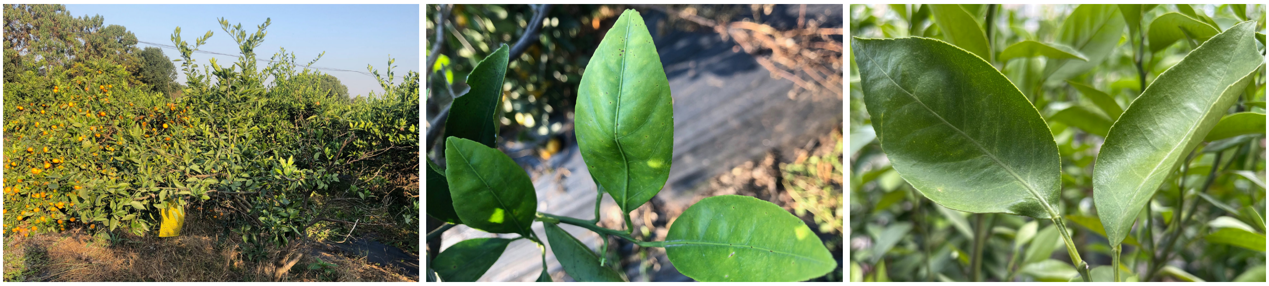

Orchard landscape | *D. citri*-infected | Uninfected

Fig. S1 The visual symptoms of *D. citri*-infected and uninfected leaves. The infected and uninfected samples were collected from a citrus orchard in in August 2019, where the melanose disease (caused by *D. citri*) had been severely occurred for three sequential years.

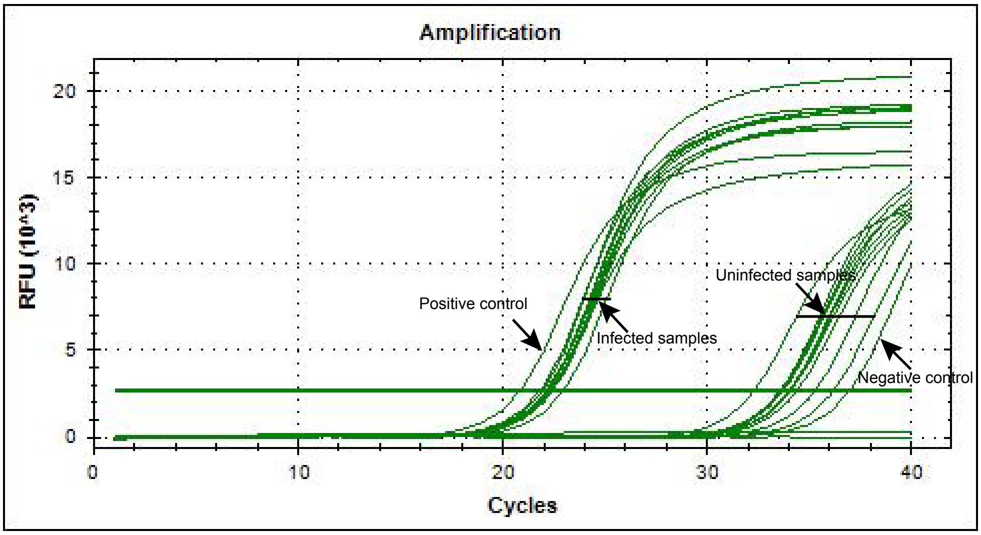

Fig. S2 qPCR amplification of melanose pathogen *D. citri*. The infected (Ct value <30) and uninfected samples (Ct value >30) were identified based on qPCR results. The β-tubulin (TUB) gene was amplified using the forward primer Dc-F (CCCTCGAGGCAT-CATTAC), and reverse primer Dc-R (ATGTTGCAGATGGTCAAATGG).

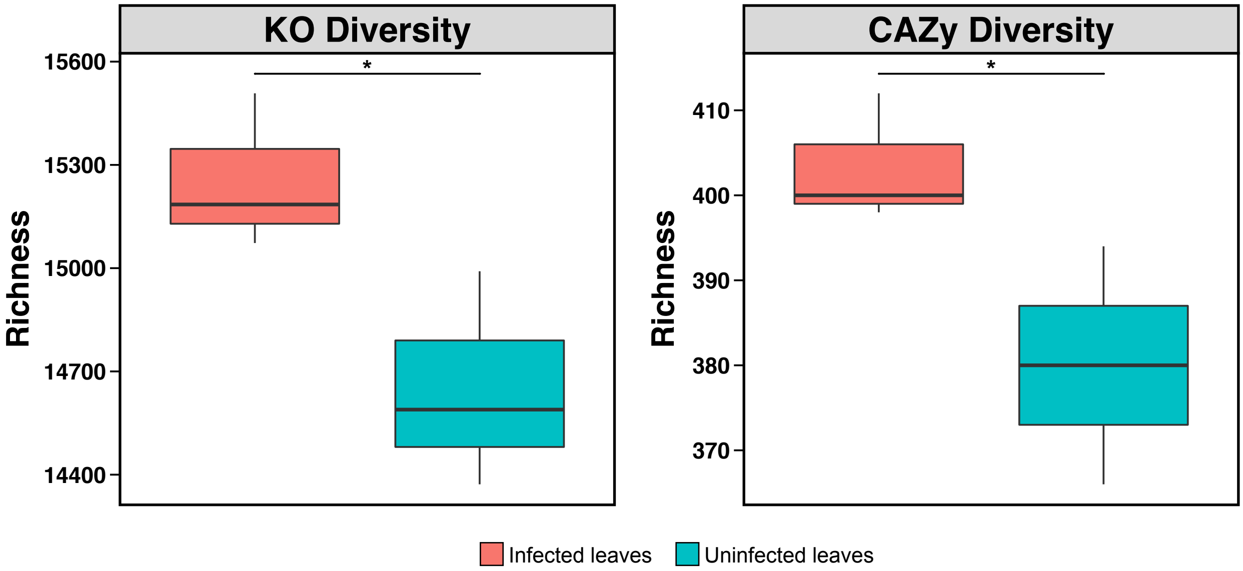

Fig. S3 Functional diversity between infected and uninfected phyllosphere microbiomes. Richness was conducted to characterize the KO and CAZy diversity and analyzed in Kruskal-Wallis to test for differences. Asterisks denote significant differences (*P <0.05; **P <0.01; ***P <0.001) and NS denotes no statistical significance.

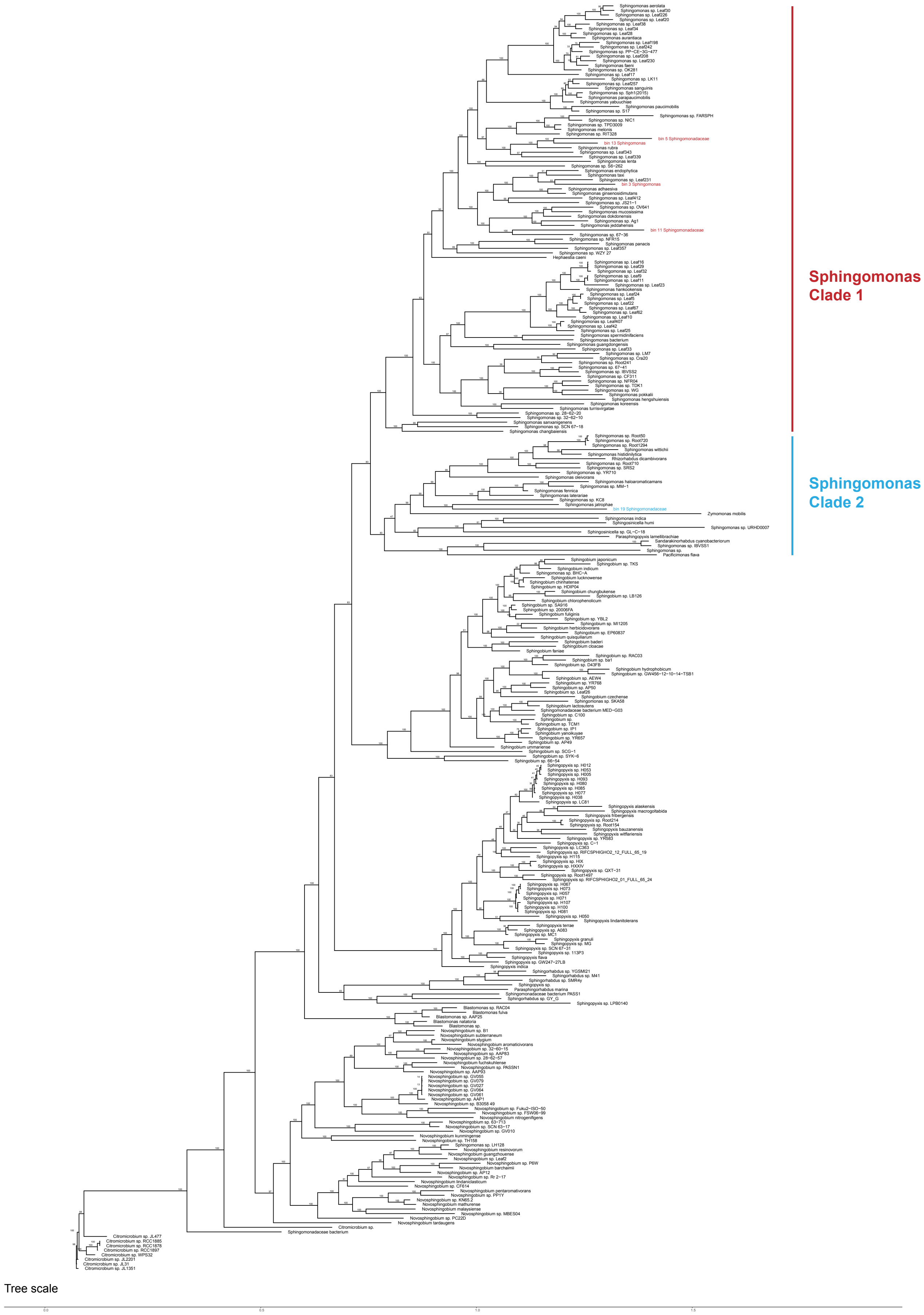

Fig. S4 A high-resolution phylogenetic tree of Sphingomonadaceae bins. 272 Sphingomonadaceae representative genomes and the bin 3, 5, 11, 13 and 19 were selected to further construct a phylogenetic tree using PhyloPhlAn.
